# Suprachoroidal Injection of Triamcinolone Acetonide Suspension: Ocular Pharmacokinetics and Distribution in Rabbits Demonstrates High and Durable Levels in the Chorioretina

**DOI:** 10.1089/jop.2021.0090

**Published:** 2022-08-01

**Authors:** Leroy Muya, Viral Kansara, Megan E. Cavet, Thomas Ciulla

**Affiliations:** ^1^ Clearside Biomedical, Inc., Alpharetta, Georgia, USA.; ^2^ Bausch + Lomb, Rochester, New York, USA.

**Keywords:** suprachoroidal, CLS-TA, triamcinolone acetonide, microinjector, microneedle, glide force

## Abstract

**Purpose:**

To compare ocular pharmacokinetics (PK), systemic absorption, and injectability of triamcinolone acetonide (TA) suprachoroidal (SC) and intravitreal (IVT) suspensions.

**Methods:**

New Zealand White (NZW) rabbits received a bilateral injection of 4 mg TA injectable suspension, TRI (TRIESENCE ^®^; Alcon) through either microneedle-based SC or standard IVT injection. Another group of NZW rabbits received a bilateral SC injection (4 mg) of either TRI or a proprietary TA suspension for SC use, CLS-TA (Clearside Biomedical). Blood and ocular tissues were analyzed for TA over 3 months. Separately, injectability of TRI and CLS-TA through a proprietary SC delivery system (SCS Microinjector^®^; Clearside Biomedical) was compared using microinjector syringe-plunger glide force measurement.

**Results:**

Suprachoroidal delivery of TRI, compared with IVT-TRI, resulted in ∼12-fold higher exposure in the retinal pigment epithelium–choroid–sclera, and comparable exposure in the retina. Conversely, SC-TRI, compared to IVT-TRI, resulted in 460-, 34-, and 22-fold lower exposure in the lens, iris-ciliary body, and vitreous humor, and negligible exposure in the aqueous humor. SC injection of either CLS-TA or TRI resulted in comparable and sustained ocular TA levels. Plasma TA levels were generally undetectable in both studies. A significantly greater and more variable glide force was measured for TRI vs. CLS-TA.

**Conclusions:**

Suprachoroidal delivery of TA enabled high and durable TA levels targeted to the chorioretina with limited anterior segment exposure. SC delivery of either CLS-TA or TRI resulted in comparable ocular PK profiles with low systemic exposure; however, CLS-TA required lower and less variable glide force than TRI, potentiating more consistent, controlled administration.

## Introduction

Macular edema (ME), the accumulation of intra- or subretinal fluid in the macular region, is a leading cause of visual impairment for many retinal diseases including uveitis, retinal vein occlusion, and diabetic retinopathy.^1–3^ Corticosteroids are often used in the treatment of ME associated with these ocular conditions.^1,4^ Delivery of corticosteroids to treat ME is currently achieved by periocular injection (transeptally or into the sub-Tenon's space), by intravitreal (IVT) injection of suspension formulations or by implantation of sustained-release drug delivery devices directly into the vitreous.^1,4,5^

Although effective, these delivery routes are often linked to high rates of corticosteroid class-associated adverse events (AEs) such as elevation in intraocular pressure (IOP)^6,7^ and cataract formation,^6,8–10^ presumably owing to diffusion of drug to anterior ocular tissues such as the aqueous humor (AH), ciliary body, and lens.^11,12^

The suprachoroidal space (SCS), a potential space between the sclera and the choroid, is a route of administration under investigation that facilitates compartmentalization of drugs,^13–15^ including corticosteroids,^16^ to the posterior segment for the treatment of vitreoretinal diseases.^17^ Suprachoroidal (SC) administration offers targeted drug delivery to the choroid, retinal pigment epithelium (RPE), and retina, while limiting drug exposure to off-target anterior tissues.^18–20^ Studies have shown that following SC injection, the administered drug flows circumferentially toward the posterior pole of the eye with delivery to the anterior tissues prevented by the scleral spur.^19–21^

The safety and efficacy of CLS-TA, a proprietary preservative-free 40 mg/mL triamcinolone acetonide (TA) injectable suspension for SC use, administered to the SCS using a proprietary microinjector device has been assessed clinically for the treatment of ME associated with noninfectious uveitis.^22–24^ In these studies, the dose (4 mg/eye) was well tolerated, significantly reduced the central subfield thickness (CST), and resulted in clinically significant improvement in vision relative to the sham injection.^22–24^ In preclinical assessments utilizing pig and rabbit models of uveitis, TA formulations administered by SC injection resulted in efficacious reduction in posterior segment inflammation^18,21^; in the pig model, 1/10th the dose of TA administered suprachoroidally was as effective as the full dose administered intravitreally.^19^

Use of the proprietary microinjector enables minimally invasive administration of this small molecule into the SCS, whereas the low aqueous solubility of TA renders it a suitable candidate for delivery in a suspension formulation with sustained release properties.^18,25–28^ During the SC procedure using the proprietary SCS Microinjector^®^ (Clearside Biomedical, Alpharetta, GA), a reduction in force required to overcome the syringe-plunger's resistance to flow^29^ is typically an indication to the clinician performing the injection procedure that the microneedle tip is in the SCS, and that the injection can successfully be completed. Lower and less variable resistance to fluid flow while the needle is in the SCS allows for more efficient, consistent, and controlled therapeutic administration.

The objectives of this research were 3-fold, i.e., (i) to assess the injectability of two TA formulations, TRIESENCE^®^ (TRI)—an FDA-approved TA injectable suspension indicated for uveitis and other ocular inflammatory conditions, and CLS-TA—a proprietary SC injectable TA suspension recently FDA approved as XIPERE^®^ for treatment of ME associated with uveitis, by determining relative microinjector syringe-plunger glide forces under conditions designed to simulate the SC injection procedure; (ii) to assess the potential for targeted posterior segment delivery of suprachoroidally administered TA by comparing ocular pharmacokinetics (PK) of TA following either microneedle-based SC injection, or standard IVT injection; and, (iii) to compare ocular PK of suprachoroidally administered TRI vs. CLS-TA.

## Methods

Studies were conducted evaluating two 40 mg/mL TA formulations, TRIESENCE (Alcon, Fort Worth, TX), and an investigational proprietary SC injectable TA suspension (Clearside Biomedical, Inc., Alpharetta, GA). TRIESENCE and the investigational injectable TA suspension are referred to as TRI and CLS-TA, respectively, in this research work.

### Injectability and microinjector syringe-plunger glide force assessment

Microinjector syringe-plunger glide force (force applied to the microinjector plunger to expel the formulation during a simulated 7 s SC injection reported in Newton (N) units) was determined for CLS-TA and TRI with 12 replicates to assess the relative injectability of CLS-TA and TRI through the SCS Microinjector. An additional 12 injections using air and water as the media were also evaluated as controls.

During each simulated SC injection, a 30G × 1,100 μm needle was attached to an SCS microinjector and injection force data were collected using a Mark-10 tensile stand with a force gauge and syringe fixture attached (Mark-10, Copiague, NY). The piston of the syringe was advanced at a linear rate of 100 mm/min, in line with the clinical administration dispense rate. Glide forces were measured as a function of time and average glide forces compared using analysis of variance (ANOVA) with *post hoc* Tukey analysis.

### *In vivo* studies

#### IACUC and compliance

All in-life procedures were conducted according to the Association for Research in Vision and Ophthalmology (ARVO) statement for the Use of Animals in Ophthalmic and Vision Research and the Institute of Laboratory Animal Resources Guide for the Care and Use of Laboratory Animals. Study protocols were reviewed and approved by the Institutional Animal Care and Use Committee for each study site before study initiation.

#### Study animals, identification, and husbandry

Male New Zealand White [Hra:(NZW)SPF] rabbits ∼10 weeks of age and weighing between 2.5 and 2.8 kg were used in the SC/IVT TRI study, and female New Zealand White [Hra:(NZW)SPF] rabbits ∼7–10 months of age and weighing between 2.5 and 4.0 kg were used in the SC CLS-TA/TRI study. A comprehensive health assessment was performed on both eyes of study animals before study initiation. A high fiber diet was fed to the rabbits throughout the acclimation and study periods according to site standard operating procedures, and water was provided *ad libitum*. Environmental controls for the animal rooms were set to maintain a temperature of 16°C to 22°C, a relative humidity range of 30%–70%, and a 12-h light/12-h dark cycle.

### Dosing

#### Suprachoroidal vs. Intravitreal TRI (Study 1)

Rabbits (*N* = 50) were randomly assigned to 2 groups based on equal body weight distribution. On the day of dosing, rabbits were anesthetized with a subcutaneous injection of ketamine/xylazine (45/5 mg/kg), followed by the application of 1 drop of proparacaine into each eye. One group received a bilateral SC injection of TRI (4 mg/100 μL/eye), and the other group received a bilateral IVT injection of TRI (4 mg/100 μL/eye).

#### SC injection

A single SC injection of 100 μL/eye was administered bilaterally over ∼5–10 s at a location 5–6 mm from the limbus, in the superior temporal quadrant using a proprietary microneedle attached to a syringe. The microneedle was held in place for ∼30 s following injection, and upon withdrawal, a cotton-tipped applicator was placed over the injection site for ∼10 s to minimize potential reflux.

#### IVT injection

A single IVT injection of 100 μL/eye was performed ∼4 mm posterior to the limbus using a 30G needle. The needle was inserted at a 45° angle at either the 11- or 1-o'clock position toward the back of the eye to avoid damaging the lens. The injection was performed over a period of ∼5 s, the needle withdrawn and as was the case for SC injection, a cotton-tipped applicator was placed over the injection site for ∼10 s to minimize potential reflux.

#### SC administration of CLS-TA vs. TRIESENCE (Study 2)

Rabbits (*N* = 20) were randomized and assigned to receive a bilateral SC injection (100 μL/eye) of either CLS-TA (40 mg/mL TA) or TRI (40 mg/mL TA). Before dosing, rabbits were anesthetized with an intramuscular (IM) injection of ketamine (45 mg/kg), dexmedetomidine (0.0315 mg/kg), and glycopyrrolate (0.01 mg/kg). An additional IM injection of flunixin meglumine (2 mg/kg) was administered as needed for analgesia. SC injection was performed as described earlier.

### Ophthalmic examinations, intraocular pressure and health

Rabbits in both studies were observed daily for any clinical and ocular changes. Biomicroscopy in study 1 was performed using a slit-lamp biomicroscope to confirm appropriate placement of the injection and in study 2, both slit-lamp biomicroscope and an indirect ophthalmoscope were used. Body weights were taken predose, weekly, and at euthanasia in both studies, whereas IOP was measured predose and on the day before and the day of scheduled euthanasia per interval in the retinal pigment epithelium using a TonoLab rebound tonometer (Icare VET, Espoo, Finland).

### Tissue collection

On designated days after dosing, study 1 animals (*n* = 5 per group [10 eyes] per time point and study 2 animals (*n* = 2 per group [4 eyes] per time point) were euthanized by intravenous overdose of sodium pentobarbital. Blood was collected using cardiac puncture and centrifuged to obtain plasma. The following ocular tissues were collected from each eye: AH, retina, RPE choroid/sclera (RCS), and vitreous humor (VH), as well as lens and iris/ciliary body (ICB) in study 1 only. All samples were stored at −80°C to −70°C until shipment for bioanalysis.

### Bioanalysis

The concentration of TA in ocular tissues and plasma was determined by liquid chromatography with tandem mass spectrometry (LC-MS/MS). An internal standard (0.025 mL of 500 ng/mL triamcinolone acetonide-d7 in DMSO) and EtOAc (1.00 mL) was added to each tissue homogenate sample. Each sample was subsequently centrifuged for 5 min, and the supernatant evaporated to dryness at 50°C under nitrogen. Calibration standards were prepared in 8 M urea with 2% SDS. A triple quadrupole mass spectrometer, Applied Biosystems SCIEX API 4000 and API 5000 for high range and low range analysis, respectively, with a TurboIonSpray source in Multiple Reaction Monitoring (MRM) mode for low range analysis was used along with an Agilent Poroshell 120 EC-C-18 column (2.1 × 50 mm, 2.7 μm particle size) operating at 50°C with mobile phase gradient of 0.1% formic acid in water and methanol.

In study 1, the quantitation range was 0.500–1,500 ng/mL for low range tissue analysis and 150–500,000 ng/mL for analysis of ocular tissues in the high concentration range. The acceptable method precision range for the calibration standards had a coefficient of variation (CV) of 2.6%–6.2% and 4.4%–9.9% for plasma and tissue homogenates, respectively. In study 2, the LC-MS/MS method used for the analysis of TA had a quantitation range of 50.0–500,000 ng/mL for tissue in the high concentration range and 0.500–1,500 ng/mL for plasma and tissues in the low concentration range. The acceptable method precision range was 2.6%–7.3% (CV) and 3.4%–11.5% (CV) for plasma and tissues, respectively.

### Pharmacokinetic and statistical analysis

Noncompartmental PK parameters C_max_ (maximum concentration) and T_max_ (the time at which C_max_ was observed) were obtained by visual inspection of the drug concentration–time curve. The area under the concentration–time curve from time “0” to the last measurable time-point (AUC_0-t_) was based on the trapezoidal rule and calculated using Phoenix WinNonlin version 6.2.1 (Pharsight Corp.). Descriptive statistics were performed using Excel^®^ or Prism^®^ (version 3.03 or 5.01; GraphPad Software, Inc., San Diego, CA).

## Results

### Injectability and microinjector syringe-plunger glide force assessment

**FIG. 1.**
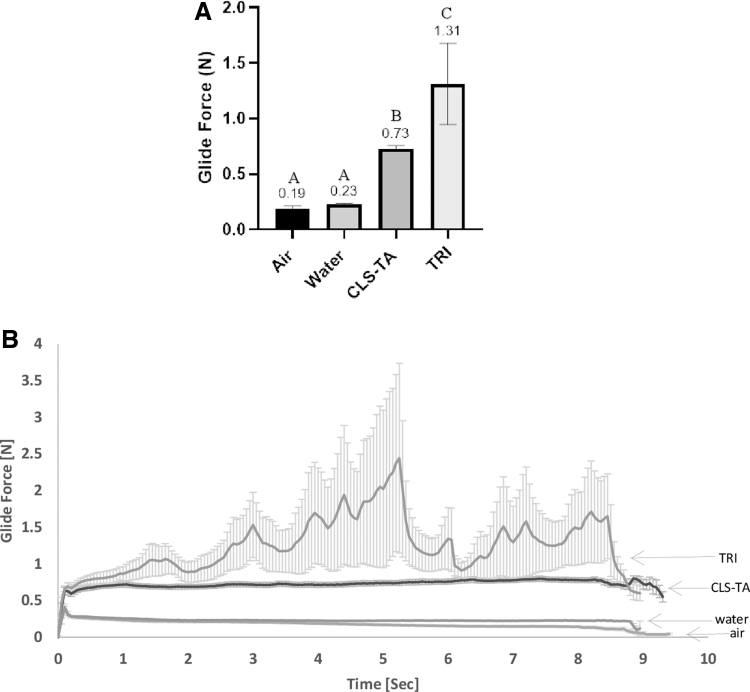
Injectability of CLS-TA and TRI under simulated SCS injection conditions **(A)** Mean (± SEM) glide force for CLS-TA and TRI during syringe compression using an SCS Microinjector^®^ and measured as average force between 1–7 s of each injection. Groups A–C denote statistically significant groups. Twelve total samples were assessed per group. **(B)** Average (mean ± SEM) glide force over time for CLS-TA and TRI during syringe compression using an SCS Microinjector (*n* = 12 per group). Error bars are present in each of the groups assessed and are standard for these types of measurements. CLS-TA, proprietary TA suspension for suprachoroidal use; TRI, TRIESENCE^®^.

Analysis of average glide forces for CLS-TA, TRI, and the air and water control samples yielded 3 distinct statistically different groups as shown in Fig. 1A. The glide force was statistically significantly higher for TRI, compared with CLS-TA (*P* < 0.001, α = 0.05). Figure 1B demonstrates the glide force of the formulations over time during dispensing and shows that TRI was also associated with higher variability in the glide force as a function of time as indicated by the high standard error.

### Ocular and systemic PK of TA after IVT or SC injection of TRI

**FIG. 2.**
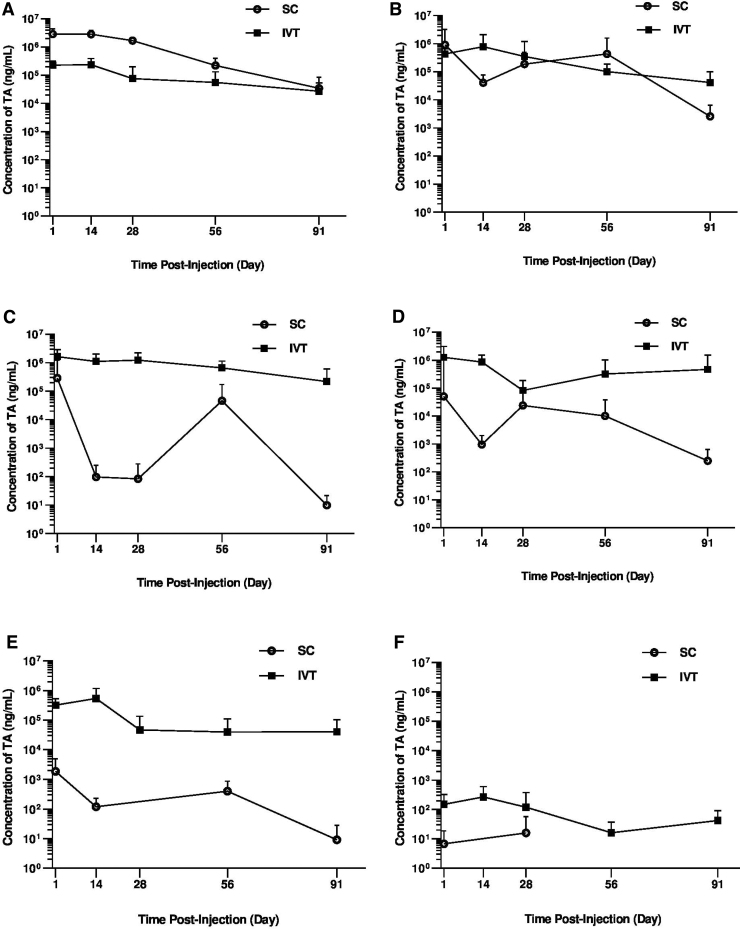
Pharmacokinetic profile of TA in rabbits. Mean (±SD) TA levels in the **(A)** RCS, **(B)** retina, **(C)** vitreous humor, **(D)** ICB, **(E)** lens, and **(F)** aqueous humor of rabbits following bilateral SC or IVT administration of 4 mg/eye TRI. *n* = 10 eyes per time point. ICB, iris/ciliary body; IVT-TRI, intravitreally administered TRIESENCE^®^; RCS, retinal pigment epithelium/choroid/sclera; SC-TRI, suprachoroidally administered TRIESENCE^®^; TA, triamcinolone acetonide.

Measurable TA levels were observed in most ocular tissues through the 91-day study after either SC or IVT administration of TRI; however, the tissue patterns of exposure differed by administration route as shown in Fig. 2A–F. Corresponding C_max_ and AUC_0-t_ values are shown in Table 1. Following SC-TRI injection, both the TA C_max_ and AUC_0-t_ values were observed in the following rank order: RCS > retina > VH > ICB > lens > AH, whereas following IVT-TRI injection, TA was observed in the rank order VH > ICB > retina > lens > RCS > AH, with the highest TA levels at the site of dose administration for both injections (Fig. 2 and Table 1).

**Table 1.** Ocular Pharmacokinetic Parameter Values for Triamcinolone Acetonide Following Bilateral Suprachoroidal Injection vs. Intravitreal Injection of 4 mg/Eye TRIESENCE in Rabbits

Tissue^1^	Injection route	C_max_ (ng/mL)		AUC_(0-t)_ (ng·day/mL)		T_max_ (days)
		Mean [SD]	SCS:IVT	Mean	SCS:IVT	
RCS	IVT	235,000 [153,000]	12.2	8,640,000	11.7	14.0
	SC	2,860,000 [1,520,000]		101,000,000		1.00
Retina	IVT	781,000 [1,320,000]	1.16	25,100,000	0.979	14.0
	SC	907,000 [2,280,000]		24,600,000		1.00
Vitreous humor	IVT	1,640,000 [1,220,000]	0.175	77,600,000	0.0445	1.00
	SC	287,000 [758,000]		3,460,000		1.00
Lens	IVT	540,000 [633,000]	0.00345	12,600,000	0.00217	14.0
	SC	1,870 [3,120]		27,400		1.00
Iris/ciliary body	IVT	1,260,000 [1,860,000]	0.0399	40,900,000	0.0291	1.00
	SC	50,200 [146,000]		1,190,000		1.00
Aqueous humor	IVT	269 [326]	0.0590	8,500	NA^a^	14.0
	SC	15.9 [41.9]		NA^a^		28.0
Plasma	IVT	3.72 [0.24]	1.23	84.2	0.94	1.00
	SC	4.59 [1.08]		78.9		1.00

^a^ AUC_0-t_ could not be calculated for aqueous humor following SCS injection because there were not at least 3 measurable concentration values.

1 *n* = 5 eyes per group per time point.

AUC_0–t_, Area under the concentration vs. time curve from the time of dosing through last time point; C_max_, maximum concentration; IVT, intravitreal; RCS, retinal pigment epithelium/choroid/sclera; SCS, suprachoroidal space; T_max_, time C_max_ was observed.

As expected, TA concentrations in the RCS (dose depot) were higher for the initial 2 months postdose for the SC vs. IVT groups with comparable TA levels at the end of the 3-month study (Fig. 2A). The mean (±SD) TA concentrations in the RCS ranged from 2,860 ± 1,520 μg/mL on day 1 to 34 ± 51.3 μg/mL on day 91, compared with lower TA levels after IVT dosing, ranging from 226 ± 93.8 μg/mL on day 1, with a C_max_ of 235 ± 153 μg/mL on day 14, to 27.1 ± 26.4 μg/mL on day 91. The SC-TRI resulted in a 12-fold higher AUC_(0-t)_, and 12-fold higher C_max_ in the RCS, compared with the IVT-TRI (Table 1).

TA levels in the retina were comparable for the SC cohort relative to the IVT cohort over the study duration (Fig. 2B), which is also evident from the calculated AUC_0-t_ (Table 1). The mean concentrations of TA levels in the retina ranged from 907 ± 2,280 μg/mL on day 1 to 2.6 ± 3.9 μg/mL on day 91 with SC dosing and from 429 ± 217 μg/mL on day 1, with a C_max_ of 781 ± 1,320 μg/mL on day 14, to 41.50 ± 58.5 μg/mL on day 91 with IVT dosing.

Overall, TA levels in the VH, ICB, lens, and AH were lower for suprachoroidally dosed animals compared with the IVT-dosed cohort over the 3-month study duration (Fig. 2C–F). In contrast to SC-TRI, which resulted in highest TA levels in the RCS, after IVT-TRI administration the highest levels of TA were measured in the VH, with mean concentrations ranging from 1,640 ± 1,220 μg/mL on day 1 to 218 ± 380 μg/mL on day 91. Vitreous humor TA concentrations for the SC-TRI cohort were highly variable, with comparatively low levels peaking at 287 ± 758 μg/mL on day 1, and with lower levels on day 14 and day 28 followed by higher levels on day 56 before declining again to 9.85 ± 12 ng/mL on day 91. The IVT-TRI resulted in ∼6-fold and 23-fold higher C_max_ and AUC values in the VH, compared with SC administration, respectively.

Levels of TA in the anterior tissues (ICB, lens, and AH) were lower after SC administration relative to the IVT administration (Fig. 2D–F), resulting in lower C_max_ and AUC values for these tissues (Table 1). A 25-fold lower C_max_ of 50.2 ± 146.0 μg/mL on day 1 compared with 1,260 ± 1,860 μg/mL on day 1 for ICB, a 289-fold lower C_max_ of 1.870 ± 3.120 μg/mL on day 1 compared with 540 ± 633 μg/mL on day 14 for lens, and a 17-fold lower C_max_ of 15.9 ± 41.9 ng/mL on day 28 compared with 269 ± 326 ng/mL on day 14 for AH, were observed for the SC- and IVT-dosed cohorts, respectively. The IVT group exhibited 460-fold and 34-fold higher AUC values in the lens and ICB, respectively, compared with the SC group, whereas a fold-increase for AH could not be calculated because TA was only detected at 2 of the time points following SC administration (Table 1).

Mean plasma concentration of TA peaked on day 1 at 4.59 ± 1.08 ng/mL and 3.72 ± 0.432 ng/mL in the SC and IVT groups, respectively, and were generally below the limit of quantitation by day 91 for both groups.

There were no observed adverse effects related to treatment or route of administration, except for 4 incidences of ocular cloudiness in the IVT group 6–7 weeks postdose which did not resolve, and no significant changes in body weight over the study period. IOP following administration of TA remained unchanged relative to baseline and between groups at each postinjection time point (Table 2).

**Table 2.** Average Intraocular Pressure Following Suprachoroidal or Intravitreal Injection of TRIESENCE

Group	No. of animals (N = 5 per group/time point)	Dose route^*^	Target dose level (mg/eye)	Average intraocular pressure [standard deviation] (mmHg)					
				Baseline	Day 1	Day 14	Day 28	Day 56	Day 91
1	25	Suprachoroidal	4	10.7 [2.0]	10.1 [2.8]	13.1 [2.7]	10.4 [1.9]	12.8 [2.2)	11.0 [1.0]
2	25	Intravitreal	4	11.0 [2.3]	9.1 [2.9]	13.3 [2.2]	12.6 [3.5]	11.2 [1.5]	11.3 [1.5]

^*^ Single bilateral injection.

### Ocular and systemic PK of TA following SC injection of either TRI or CLS-TA

**FIG. 3.**
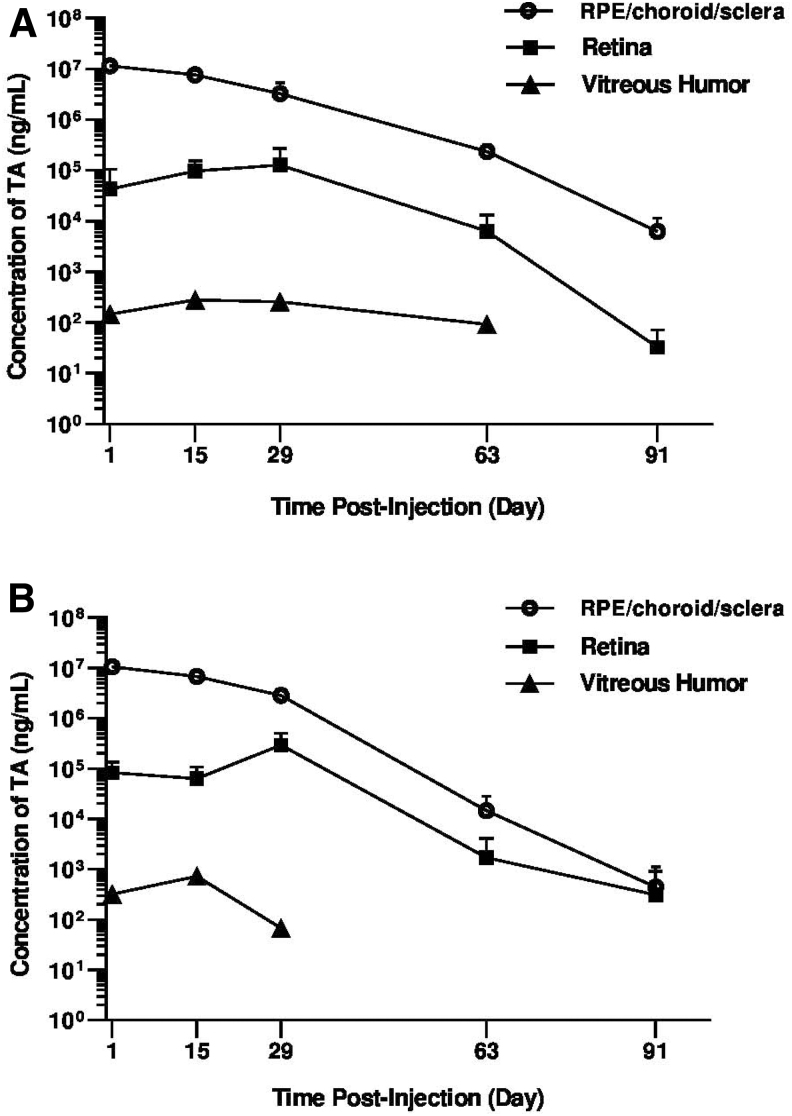
Mean (±SD) concentrations (ng/mL) of TA in rabbit ocular tissues following bilateral suprachoroidal injection of **(A)** 4 mg/eye CLS-TA and **(B)** 4 mg/eye TRI. *n* = 4 eyes per time point. RPE, retinal pigment epithelium; TA, triamcinolone acetonide; CLS-TA, proprietary TA suspension for suprachoroidal use; TRI, TRIESENCE.

Following SC administration of TRI and CLS-TA, the TA C_max_ in the ocular tissues were in the following rank order: RCS > retina > VH (Table 3), whereas no TA was detected in the AH throughout the study duration for either formulation. Comparable high TA levels were detected in the RCS after SC injection of either CLS-TA or TRI, with a mean C_max_ of 11.6 ± 0.64 mg/mL and 10.7 ± 0.52 mg/mL at day 1 postdose, and an AUC of 273 mg·day/mL and 242 mg·day/mL, respectively. Similar TA levels were detected in the retina for both formulations throughout the study with retinal levels declining to 0.0326 ± 0.0385 μg/mL and 0.313 ± 0.598 μg/mL on day 91 for CLS-TA and TRI, respectively (Fig. 3A, B). A mean C_max_ of 129 ± 142 μg/mL and 294 ± 216 μg/mL on day 29, and mean AUC values of 4,860 μg·day/mL and 7,870 μg·day/mL were observed in the retina, for CLS-TA and TRI, respectively (Table 3).

**Table 3.** Ocular Pharmacokinetic Parameter Values for Triamcinolone Acetonide Following Bilateral Suprachoroidal Injection of 4 mg/eye CLS-TA vs. TRIESENCE in Rabbits

Tissue	TA formulation	C_max_ (ng/mL)		AUC_(0-t)_ (ng·day/mL)		T_max_ (days)
		Mean [SD]	CLS-TA:TRI	Mean (SE)	CLS-TA:TRI	
Sclera/choroid	CLS-TA	11,600,000 [635,000]	1.08	273,000,000	1.12	1.00
	TRI	10,700,000 [519,000]		242,000,000		1.00
Retina	CLS-TA	129,000 [142,000]	0.43	4,860,000	0.62	29.0
	TRI	294,000 [216,000]		7,870,000		29.0
Vitreous humor	CLS-TA	281 [441]	0.38	12,800	0.98	15.0
	TRI	733 [1,300]		13,100		15.0
Plasma^#^	CLS-TA	4.22	0.88	65.2	0.83	1.00
	TRI	4.82		78.8		1.00

# Plasma data were *n* = 2 and therefore the SD was not calculated.

CLS-TA, proprietary TA suspension for suprachoroidal use; TRI, TRIESENCE^®^.

Limited TA exposure to the VH was observed following SC injection of either TA formulation, with mean C_max_ of 0.281 ± 0.441 μg/mL and 0.733 ± 1.3 μg/mL on day 15, and AUC values of 12.8 μg·day/mL and 13.1 μg·day/mL, for CLS-TA and TRI, respectively. No TA was detected in the AH throughout the study duration for either formulation. Plasma C_max_ values of 4.22 ng/mL and 4.82 ng/mL were reached on day 1 for CLS-TA and TRI, respectively (Table 3), and concentrations decreased rapidly through the end of the study where TA levels were below levels of quantitation.

In general, SC injections of both TA formulations (CLS-TA and TRI) were well tolerated, with a low incidence of adverse effects. Two animals in the TRI group had small focal subretinal hemorrhages in the superior-temporal quadrant of the fundus on day 8 and there was sporadic incidence of mild conjunctival hyperemia in both groups.

## Discussion

It is well known that corticosteroids inhibit inflammation, vascular leakage, and angiogenesis, but their use has been limited somewhat by their equally well-known class-associated AE profile including increased IOP and cataract formation.^11,30–32^ IVT administration is a standard and recommended route of administration of corticosteroids to uveitic patients; however, a key limitation of IVT administration is that drug exposure to unaffected ocular tissues leads to a significant risk for drug-related off-target adverse effects.^11,32^

SC delivery targets and compartmentalizes drugs to the posterior segment while minimizing exposure to the anterior segment. Access to the SCS can be achieved by a variety of methods including the use of a catheter, a standard hypodermic needle, or a microinjector with a microneedle attached. In the case of the proprietary SCS Microinjector, 2 microneedle lengths of 900 μm and 1,100 μm are optimized for anatomic variation in human subjects. These lengths take into account the scleral and conjunctival thickness at the pars plana to ensure that the needle penetrates through the scleral layer to deliver drug to the potential space between the sclera and the choroid, while preventing penetration into the vitreous as would be the case for IVT delivery, which utilizes a standard hypodermic needle and syringe.^19,33^

Glide force, an integral attribute contributing toward successful injection when using the proprietary microinjector, was assessed comparing CLS-TA and TRI. The average CLS-TA glide forces were statistically lower (0.73 N) and less variable compared with glide forces required for injections using TRI (1.31 N) under the same simulated conditions. Key factors that may play a role in the differing glide forces and degree of variability in glide forces between drug products during simulated injections of the same active pharmaceutical ingredient (API), in this case TA, are particle size, as well as the choice and concentration of excipients. Tightly controlled particle size distribution is critical in ensuring consistent drug product properties, lending to consistency in drug product performance impacting PK profiles when administered for therapy.

In the case of excipients, whereas a wide array including viscosity enhancing agents and surfactants are approved for ophthalmic applications,^34^ the concentrations and physicochemical properties of each chemical entity utilized can significantly impact the drug product's performance during administration using a microneedle/injector system. Surfactants, often used to improve solubility of APIs and promote homogeneous re-dispersibility by reducing surface tension contributions from their hydrophobic moieties,^35,36^ also play multiple roles in improving wetting, reducing coefficient of friction, minimizing foaming and formation of microbubbles—all of which may directly impact both the microinjector syringe-plunger glide force and degree of variability of these glide forces during the injection procedure.^37,38^

In general, lower injection forces are expected to translate to a clearer tactile perception of loss of resistance during the SC injection procedure, a signal to the injecting clinician that the microneedle tip is in the SCS, and that the injection can successfully be completed. Crucially, failure to detect this loss of resistance signal also determines the necessity for changing to the longer 1,100 μm needle. Lower and less variable glide force data support the development of CLS-TA suspension formulation for SC delivery and its use over TRI for more efficient, consistent, and controlled SC administration using the proprietary microneedle and microinjector.

In these investigations, TA was delivered to the SCS of rabbit eyes through a proprietary microneedle. In the first study, suprachoroidally administered TA was distributed primarily in the RCS (dose depot) and retina, whereas IVT-administered TA distributed primarily to the VH and throughout the eye, including the anterior tissues. Furthermore, in the second study, SC administration of 2 different TA formulations, TRI and CLS-TA, in rabbits, also resulted in distribution of TA primarily in the chorioretina and sclera with limited exposure to the VH. Of note, no TA was detected in the AH in this study, providing evidence that SC administration limits exposure to the anterior segment of the eye.

Together these data demonstrate that directly targeting the SCS for delivery of therapeutic agents results in high drug bioavailability in the choroid and other posterior segment tissues, while limiting levels of drug exposure to anterior segment tissues including the lens and ICB, thereby minimizing the potential incidence and degree of cataract formation as well as other AEs associated with IVT corticosteroid administration. High levels of TA were detectable in the posterior tissues, including the dose depot (RCS) and retina, for the entire duration of the 3-month study, following SC injection, thereby facilitating prolonged durability of action of the drug. These data, along with higher AUC values detected in the dose depot for the SC-dosed group relative to the IVT-dosed group also suggest the potential for improved and/or longer action in the chorioretina, although head-to-head pharmacodynamic studies are needed.

Clinical study data align with the findings of the two PK rabbit studies. The safety and efficacy of CLS-TA administered via the SC route has been evaluated in a phase 3 double-masked randomized clinical trial (PEACHTREE).^22^ In this study involving 160 patients with ME secondary to noninfectious uveitis receiving 2 doses of CLS-TA (4 mg/eye) 12 weeks apart (at baseline and at week 12), mean retinal CST was significantly reduced as early as at 4 weeks postinjection (the earliest assessment time point) and remained reduced through 6 months indicating SC delivery of CLS-TA resulted in therapeutic levels of TA in chorioretinal tissues. In addition, 47% of subjects showed a meaningful 15 Early Treatment Diabetic Retinopathy Study (ETDRS) letter improvement in best corrected visual acuity (BCVA) from baseline compared with 16% in the sham-control group at 24 weeks (*P* < 0.01).

In the MAGNOLIA extension study, the duration of action of CLS-TA was assessed as a measure of time to rescue and was evaluated over an additional 24 weeks following exit from PEACHTREE. In the MAGNOLIA study, none of the randomized patients in the CLS-TA arm were to receive any protocol-mandated additional CLS-TA. Fifty percent (50%) of CLS-TA patients in the MAGNOLIA study did not require rescue therapy for ∼9 months after receiving the second SC injection in PEACHTREE. Patients in the CLS-TA group that did not require rescue were also reported to have retained their reductions in CST and improvements in ETDRS letters read through the end of the MAGNOLIA study.^39^

Finally, in the open-label, phase 3 safety trial (AZALEA),^24^ 38 subjects with noninfectious uveitis with or without macula edema received 2 CLS-TA (4 mg) injections 12 weeks apart (at baseline and week 12). Safety and efficacy parameters were assessed including changes in signs of inflammation, BCVA, and retinal CST. No serious AEs were reported during the study. Suprachoroidally administered CLS-TA was well tolerated and efficacy parameters improved over the 24-week study period. The AZALEA study also included an assessment of systemic TA levels following CLS-TA administration; TA levels detected in plasma were low (<1 ng/mL) over the 24-week study,^24^ in agreement with the minimal systemic exposure following SC administration of either TRI or CLS-TA observed in the rabbit PK studies.

Overall, the current PK data demonstrate that the SC route of administration provides targeted and compartmentalized delivery of drug to the posterior ocular tissues while limiting anterior segment exposure compared with IVT delivery. These ocular distribution and PK characteristics provide the potential for durable efficacy while minimizing the corticosteroid class-associated AEs of increased IOP and cataract formation that have previously been reported with other routes of corticosteroid administration.^4,40^ Clinical study data with triamcinolone acetonide injectable suspension, CLS-TA, to date support these findings; suprachoroidally injected TA resulted in improved anatomic and functional outcomes versus the control and appeared well-tolerated with a low incidence of cataract and IOP elevations. Further clinical studies exploring more molecules of interest delivered via the SC route are warranted.
